# COVID-19 during Gestation: Maternal Implications of Evoked Oxidative Stress and Iron Metabolism Impairment

**DOI:** 10.3390/antiox11020184

**Published:** 2022-01-18

**Authors:** Jorge Moreno-Fernandez, Julio J. Ochoa, Catalina De Paco Matallana, Africa Caño, Estefania Martín-Alvarez, Javier Sanchez-Romero, Juan M. Toledano, Maria Puche-Juarez, Sonia Prados, Susana Ruiz-Duran, Lucia Diaz-Meca, María Paz Carrillo, Javier Diaz-Castro

**Affiliations:** 1 Department of Physiology, Faculty of Pharmacy, Campus Universitario de Cartuja, University of Granada, 18071 Granada, Spain; jorgemf@ugr.es (J.M.-F.); juanmatd97@correo.ugr.es (J.M.T.); mpuchej@correo.ugr.es (M.P.-J.); javierdc@ugr.es (J.D.-C.); 2 Institute of Nutrition and Food Technology “José Mataix Verdú”, University of Granada, 18071 Granada, Spain; 3 Clinical Medicine and Public Health Ph.D. Program, University of Granada, 18071 Granada, Spain; 4 Department of Obstetrics and Gynecology, Hospital Clínico Universitario ‘Virgen de la Arrixaca’, El Palmar, 30120 Murcia, Spain; javier.sanchez14@um.es (J.S.-R.); ldm460@hotmail.com (L.D.-M.); 5 Institute for Biomedical Research of Murcia, IMIB-Arrixaca, El Palmar, 30120 Murcia, Spain; 6 Department of Obstetrics and Gynaecology, San Cecilio Universitary Hospital, 18071 Granada, Spain; africacano59@gmail.com (A.C.); sonigine@hotmail.com (S.P.); 7 Unit of Neonatology, Pediatric Service, Hospital Universitario Materno-Infantil Virgen de las Nieves, 18014 Granada, Spain; estefaniamartin82@gmail.com; 8 Nutrition and Food Sciences Ph.D. Program, University of Granada, 18071 Granada, Spain; 9 Department of Obstetrics & Gynaecology, Virgen de las Nieves University Hospital, 18014 Granada, Spain; sruizduran@gmail.com (S.R.-D.); mpazcb@gmail.com (M.P.C.); 10 Instituto de Investigación Biosanitaria (IBS), 18012 Granada, Spain

**Keywords:** COVID-19, placenta, pregnancy, antioxidant system, oxidative stress

## Abstract

COVID-19 has reached pandemic proportions worldwide, with considerable consequences for both health and the economy. In pregnant women, COVID-19 can alter the metabolic environment, iron metabolism, and oxygen supply of trophoblastic cells, and therefore have a negative influence on essential mechanisms of fetal development. The purpose of this study was to investigate, for the first time, the effects of COVID-19 infection during pregnancy with regard to the oxidative/antioxidant status in mothers’ serum and placenta, together with placental iron metabolism. Results showed no differences in superoxide dismutase activity and placental antioxidant capacity. However, antioxidant capacity decreased in the serum of infected mothers. Catalase activity decreased in the COVID-19 group, while an increase in 8-hydroxy-2’-deoxyguanosine, hydroperoxides, 15-FT-isoprostanes, and carbonyl groups were recorded in this group. Placental vitamin D, E, and Coenzyme-Q10 also showed to be increased in the COVID-19 group. As for iron-related proteins, an up-regulation of placental DMT1, ferroportin-1, and ferritin expression was recorded in infected women. Due to the potential role of iron metabolism and oxidative stress in placental function and complications, further research is needed to explain the pathogenic mechanism of COVID-19 that may affect pregnancy, so as to assess the short-term and long-term outcomes in mothers’ and infants’ health.

## 1. Introduction

Novel coronavirus infection (COVID-19) is caused by SARS-CoV-2. The first case was reported in December 2019, and since then the disease has quickly spread worldwide reaching pandemic dimensions. The infection typically occurs in most patients with manifestations that include fever, cough, fatigue, shortness of breath, and frequent pneumonia. Estimates from the World Health Organization (WHO) indicate that global mortality rate ranges between 3–4%, with a high proportion of patients requiring admission to intensive care units. This results in high economic costs, related to increased investment in health care and restrictions that affect circulation and trade [1].

Pregnancy presents characteristics that make pregnant women more susceptible to respiratory diseases and the pneumonia development, this being one of the most prevalent non-obstetric infections in pregnant women [2]. These characteristics are associated with some changes that include: increased oxygen consumption, elevated diaphragm, and airway edema, which makes pregnant women much more intolerant to hypoxia. This elevated susceptibility was observed during the H1N1 (Influenza A) outbreak in 2009, in which pregnant women were four times more likely to be admitted to hospital than the rest of the population [3]. In the event that a pregnant woman gets a serious respiratory infection, the most frequent complications are: premature rupture of membranes, intrauterine growth restriction, premature delivery, and fetal death [4].

Due to the rapid expansion of the pandemic, its health and economic implications, and the increased susceptibility for pregnant women to suffer its consequences, it is necessary to conduct deeper research on the effects of the virus during pregnancy and the prediction of possible adverse outcomes in maternal and fetal health. To date, although there have been several studies on COVID-19 during pregnancy, most of the clinical studies available only include a few cases (due to the low cumulative incidence among this group of population), and all of them report on routine parameters, which shows a lack of knowledge of the implications of infection during the gestational process and its sequels in maternal-fetal health [5,6].

The placenta constitutes the active interface between the maternal and fetal blood circulations, regulating physiological changes in mother and fetus, and playing a key role in the development of many pregnancy complications. This organ maintains fetal homeostasis by performing a wide range of physiological functions, which after birth, are carried out by the kidney, gastrointestinal tract, lungs, and endocrine glands of the newborn. In pregnant women, COVID-19 can alter the oxygen supply of trophoblastic cells [7], thus affecting placental function. Moreover, oxidative stress caused by this infection is able to induce direct tissue damage, including in placenta, which would negatively affect the gestation process [8,9], especially in situations of great oxidative aggression such as pregnancy and childbirth [10].

On the other hand, iron plays a crucial role in pregnancy and fetal development [11] and can be altered by the hepcidin-like activity of SARS-CoV-2. This virus induces significant dysregulation on iron metabolism, with high levels of ferritin and ferroptosis [12], which could compromise the oxygen transport to trophoblasts and induce several cell impairments [13]. In addition, hepcidin-induced hypoferremia induced by SARS-CoV-2 impairs primary and memory responses to immunization, and the high levels of hepcidin inhibits adaptive immune responses to pathogens, indicating that serum iron, regulated by hepcidin, is an important and potentially targetable control point for immunity [14]. The pro-inflammatory state associated to iron homeostasis dysregulation plays a key role in pathogenesis of disease and the hyper-ferritinemia due to SARS-CoV-2 being associated with iron toxicity because of ferritin leakage and free iron released by damaged tissues. Therefore, iron metabolism should be investigated in COVID-19 patients to monitor the clinical course of the disease, to predict negative prognosis [15].

Nevertheless, in spite of the importance of oxidative stress and iron metabolism in fetal development, there are just a few studies available in the scientific literature about COVID-19 in pregnant women, and they record just some clinical manifestations generally near late preterm and delivery [16,17], without elucidating the crucial role of both factors during pregnancy and its maternal-fetal implications. Taking into account all that mentioned above, the purpose of this study was to investigate, for the first time, the effects of COVID-19 during pregnancy on the oxidative/antioxidant status and iron metabolism, an unexplored scientific field with noteworthy clinical and social implications.

## 2. Materials and Methods

### 2.1. Subjects

**Figure 1:**
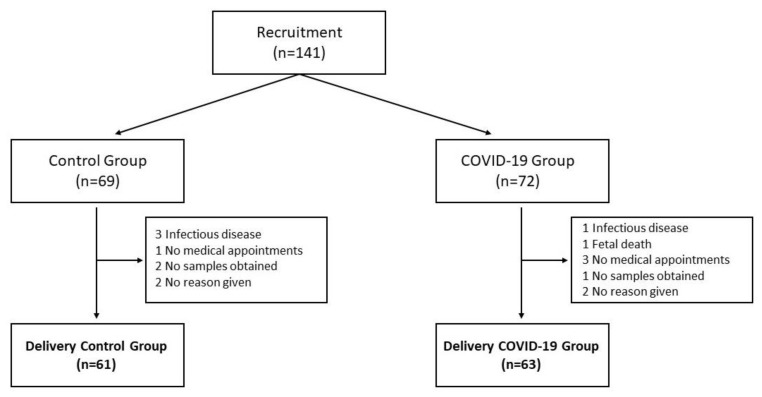
Flow chart showing the progress and abandonment of study subjects.

One hundred and twenty-four pregnant mothers were recruited from three hospitals; Hospital Materno-Infantil Virgen de las Nieves (Granada, Spain), Hospital Universitario Clínico San Cecilio (Granada, Spain), and Hospital Universitario Virgen de la Arrixaca (Murcia, Spain), between June 2020 and August 2021. Flow diagram for subject recruitment and abandonment is shown in Figure 1. Pregnant women without previous chronic pathologies were recruited in the corresponding consultation service and followed up during the rest of the pregnancy. Inclusion criteria were: freely accepting to participate in the study and having the informed consent signed by the volunteer, pregnant woman with a regular ongoing pregnancy, body mass index of 18–30 kg/m^2^ in early pregnancy, and having suffered COVID-19 (PCR+, variant Alpha, B.1.1.7 from week 28th onwards for the COVID-19 group and PCR−, not having suffered the infection during the gestation, for the control group). The exclusion criteria were: chronic disease that requires long-term treatment, infectious diseases, body mass index of <18 or >30 kg/m^2^, malnutrition, chromosome/congenital malformations, fetal death, and non-acceptance of informed consent to participate in the study. Once the nature and purpose of the study were in detail explained to them and they accepted the informed consent, the mothers were assigned to one of the study groups. All the infected mothers who participated in the study reported mild symptoms, not being asymptomatic nor requiring hospital stay. The study has been approved by the Human Research Committee of the Biomedical Research Ethic Portal of Andalucía (reference 10/20–31 December 2020).

Taking into account our main objective, and in accordance with the results obtained in a study in pregnant women in which some gestational parameters similar to the present study were determined (although not in mothers suffering from COVID-19, given the scarcity of studies in the scientific literature), 55 mothers per group were needed, taking into account the loss of approximately 20% of the population during the study [18]. To increase the statistical power and taking into consideration the prevalence of the infection in the Spanish population, we enrolled the following groups: Control Group (*n* = 61): pregnant women who have not suffered COVID-19 during pregnancy; and COVID-19 group (*n* = 63): pregnant women who have suffered COVID-19 during pregnancy (from week 28th onwards). Throughout the delivery, clinical analytical estimators were controlled and simultaneously, the necessary samples were collected for subsequent analysis. Participants were free to withdraw from the study at any time in accordance with the Declaration of Helsinki.

### 2.2. Blood Sampling

For measurements, a maternal blood sample (5.0 mL) was collected at the time of delivery in a serum vacutainer tube with separating gel. After 30 min at room temperature, the blood was centrifuged (3000× *g* rpm during 10 min at room temperature) to obtain serum. Serum samples were frozen (−80 °C) until further measurements.

### 2.3. Placenta Sampling

The placenta’s size, shape, consistency, and completeness were assessed, as well as the presence of accessory lobes, placental infarcts, bleeding, tumors, and nodules. A cut was made to detach the umbilical cord from the placenta shortly after. The leftover umbilical tissues were then discarded after the umbilical artery was severed. A sample of placental cotyledons measuring 2 cm × 2 cm × 2 cm was also taken, omitting placental membranes. Samples were removed from the center of the placenta, avoiding necrosis, infarction, and calcification. They were independently exposed to different washings with a chilled cold 0.9% NaCl solution with 0.1% butylated hydroxytoluene (BHT) (Sigma, St Louis, MO, USA) and 1 mM Ethylenediaminetetraacetic corrosive (EDTA) (Sigma). Washings were repeated until no remaining blood was distinguished. The complete length of preparation time was under 15 min. Placenta sections were frozen in liquid nitrogen and stored at −80 °C for subsequent analysis of vitamin and mineral content, and protein expression. Furthermore, fractions of homogenate were obtained from fresh placenta within the same reception day, through successive differential centrifugations with hypotonic hemolysis, according to the Hanahan and Ekholm method (1974) [19], storing these fractions at −80 °C for following analysis of antioxidant capacity, antioxidant enzymes activity, protein oxidation (carbonyl groups), 8-hydroxy-2′-deoxyguanosine (8-OHdG), 15-F2t-isoprostanes, and lipid hydroperoxides.

### 2.4. Antioxidant Capacity

Antioxidant capacity was measured in placenta and serum through ABTS (2,2’-azino-bis (3-ethylbenzothiazoline-6-sulfonic acid) assay following the method described by Re et al., (1999) [20]. A total of 7 mM ABTS stock solution was prepared and left stirring for 19 h, preserved from light. A total of 7 mM Trolox solution (6-hydroxy-2,5,7,8 Tetramethylchroman-2-carbonane 97%) and phosphate buffer saline solution (PBS) 1:10 were also prepared. Samples were diluted, ABTS was added, and finally, absorbance was measured on a microplate reader (Bio-tek, Winooski, VT, USA) at 743 nm.

### 2.5. Antioxidant Enzymes Activity

Superoxide dismutase (SOD) activity was assayed in placenta and serum according to the method of Crapo et al., (1978) [21]. This method is based on the reduction of cytochrome c inhibition by SOD, spectrophotometrically measured (Thermo Spectronic, Rochester, NY, USA) at 550 nm wavelength. Catalase (CAT) activity was determined according to Aebi method (1984) [22], spectrophotometrically monitoring at 240 nm (Thermo Spectronic, Rochester, NY, USA) the H_2_O_2_ decomposition by catalytic activity of CAT.

### 2.6. Protein Oxidation (Carbonyl Groups) Measurement

Placenta and serum protein oxidation was measured according to a method based on the spectrophotometric detection of the reaction of 2,4-dinitrophenylhydrazine (DNPH) with protein carbonyl (PC) to form protein hydrazones [23]. A total of 10 mM DNPH in 2.5 M HCl was used to treat the extracted protein. The resulting precipitates were dissolved in 2 mL of 6 M guanidine hydrochloride solution and incubated at 37 °C for 10 min. Because 10–15% of proteins were lost during the various washing phases, a procedure was developed to evaluate protein levels in final pellets. Pellets made from 2.5 M HC1-treated samples were dissolved in 6 M guanidine hydrochloride, and proteins were measured using a 280 mm absorption spectrophotometer (Bio-tek, Winooski, VT, USA). The highest absorbance (366 nm) of the DNPH-treated samples was used to compute the PC level.

### 2.7. 8-Hydroxy-2′-deoxyguanosine (8-OHdG)

8-hydroxy-2′-deoxyguanosine (8-OHdG) was measured using an in vitro enzyme-linked immunosorbent assay (ELISA) for quantitative detection of the oxidative DNA adduct 8-OHdG. To separate interfering substances, filtration of serum and placental homogenate using an ultra-filter (cut off molecular weight 10,000) was done. Results were read at 450 nm on a microplate reader (Bio-tek, Winooski, VT, USA).

### 2.8. 15-F2t-Isoprostanes

A commercial kit Enzyme Immunoassay for Isoprostane (Oxford Biomedical Research, Oxford, UK) was used to quantify isoprostanes in placenta and serum. Non-specific binding interference was virtually eliminated by mixing samples with an improved dilution buffer. The 15-F2t-Isoprostane in samples and standards competes for binding to a polyclonal antibody specific for 15-F2t-Isoprostane coated on the microplate with 15-F2t-Isoprostane conjugated to horseradish peroxidase (HRP). When substrate is added, the HRP activity causes color development, with the intensity of the color being proportional to the amount of 15-F2t-Isoprostane-HRP bound and inversely related to the quantity of unconjugated 15-F2t-Isoprostane in the samples or standards. The plate was spectrophotometrically read (Bio-tek, Winooski, VT, USA) at 450 nm.

### 2.9. Lipid Hydroperoxides

Lipid hydroperoxides levels were measured in placenta and serum either through calculation from known extinction coefficient of XO-Fe complex, or by reference from a standard curve prepared with a H_2_O_2_ solution, using the commercial kit Pierce™ Quantitative Peroxide Assay Kit (Thermo Scientific, Rockford, IL, USA).

### 2.10. Antioxidant Vitamins

Fat soluble antioxidants in placenta and plasma (vitamin D, E, A, and Coenzyme Q10) were determined. The used equipment was an ACQUITY UPLC H-Class detector coupled to a triple quadrupole Xevo TQ-S (Waters Corporation, Milford, CT, USA). All the parameters studied were individually optimized using standard solutions from Sigma-Aldrich (minimum 98% purity, Grade HPLC) and quantified with standard curves. MassLynx 4.1. (Waters Corporation, Milford, CT, USA) software was used to obtain all the data.

### 2.11. Multielemental Analysis by Inductively Coupled Plasma-Mass Spectrometry (ICP-MS)

Placenta samples were previously mineralized by wet method in a sand bath (J.R. Selecta, Barcelona, Spain); they were placed in a resistant flask and dissolved using nitric acid followed by a mixture of HNO_3_:HClO_4_ (69%:70%, *v*/*v*; Merck KGaA, Darmstadt, Germany; ratio 1:4, *v*/*v*) until complete elimination of organic matter. Fe, Mn, Se, Ba, Cu, Zn were determined using an Agilent 8800 ICP-MS (Agilent, Santa Clara, CA, USA). All ICP-MS standards were prepared from ICP single element standard solutions (Merck KGaA) after appropriate dilution with 10% HNO_3_. For calibration, two sets of multielement standards containing all the analytes of interest at five levels concentrations were prepared using Rhodium as internal standard.

### 2.12. Western Blotting and Immunocytochemistry

Proteins were extracted from placenta samples through mechanic homogenization in tissue protein extraction reagent (T-PER) (Thermo Scientific Inc., Hanover Park, IL, USA) combined with a protease inhibitor cocktail (Sigma-Aldrich, St. Louis, MO, USA) at 4 °C. A total of 12 µg of total protein were loaded on 4–20% CriterionTGX (Tris-Glycine extended) gels (Mini-PROTEAN TGX Precast Gels; Bio-Rad, Hercules, CA, USA) for separation by electrophoresis (Mini-PROTEAN System; Bio-Rad) at 250 V for 20 min. Proteins were transferred from gels to a polyvinylidene difluoride membrane (Bio-Rad) via wet transfer at 120 V for 60 min. Afterwards, electrotransferred membranes were blocked for 1 h at room temperature with 5% non-fat dry milk in Tris-buffered saline (TBS) with Tween-20 (TTBS) (Bio-Rad) solution. Membranes were washed three times in TBS and incubated with primary antibodies overnight at 4 °C and with shaking: rabbit anti-DMT1 polyclonal, dilution 1:250 (Santa Cruz Biotechnology Inc., Santa Cruz, CA, USA), rabbit anti-SLC40A1 polyclonal antibody (FPN1), dilution 1:700 (Abcam, Cambridge, UK), anti-ferritin monoclonal antibody (Abcam, Cambridge, UK), dilution 1:3000, and mouse anti-actin monoclonal, dilution 1:1000 (Abcam, Cambridge, UK) as primary antibodies. Anti-DMT1 and anti-SLC40A1 were diluted in 5% non-fat dry milk in TTBS, while anti-ferritin and anti-actin were diluted just in TTBS (being the one used as the loading control). After that, membranes were washed three times in TTBS, and incubated with the appropriate secondary conjugated antibody for 1 h at room temperature: Immun-Star Goat Anti-Mouse (GAM)-HRP, dilution 1:80,000; and Immun-Star Goat Anti-Rabbit (GAR)-HRP (Bio-Rad), dilution 1:40,000, in TTBS. Finally, the labeling reaction was detected and the intensity of the bands was visualized with a chemiluminescence Luminata forte western HRP Substrate (Merck KGaA, Darmstadt, Germany), using ImageQuant LAS 4000 (Fujifilm Life Science Corporation, Cambridge, MA, USA). Image J software was used to analyze results.

### 2.13. Statistical Analysis

All variables were tested for normality and homogenous variance using the Kolmogorov–Smirnoff and Levene tests, respectively, prior to any statistical analysis. When the data were not normally distributed, non-parametric tests (U-Mann–Whitney with non-paired samples and Wilcoxon with paired samples) were used to examine differences between groups; when the data were regularly distributed, the *t*-test for independent samples was used. SPSS software version 27.0, 2020 was used for all statistical analyses (SPSS Inc., Chicago, IL, USA).

## 3. Results

Table 1 shows the clinical characteristics (including the haematological and biochemical parameters measured in the hospital) of the mothers participating in this study. No statistically significant differences were observed between groups for age, weight, height, BMI, parity, or delivery method. In the biochemical parameters shown, differences were only found in serum Fe concentrations with lower values in the COVID-19 group compared to the control group (*p* < 0.01).

With regard to the antioxidant systems studied (Table 2), results showed no differences in SOD and antioxidant capacity of placenta, while total antioxidant capacity decreased in serum of mothers suffering from COVID-19 (*p* < 0.05). On the other hand, CAT activity decreased in the COVID-19 group (*p* < 0.01 in placenta and *p* < 0.05 in serum), while an increase in 8-hydroxy-2’-deoxyguanosine (8-OHdG) (*p* < 0.01 for placenta and serum), hydroperoxides (*p* < 0.001 for placenta), 15-FT-isoprostanes (*p* < 0.01 for placenta and serum), and carbonyl groups (*p* < 0.001 for placenta and *p* < 0.05 for serum) were recorded in the COVID-19 group.

Table 3 summarizes antioxidant vitamins in placenta and serum. In placenta, Vitamin D, E, and Coenzyme Q10 (CoQ10) showed to be increased in the COVID-19 group (*p* < 0.01 for vitamin D and E; *p* < 0.05 for CoQ10. In serum, only vitamin E levels showed an increase (*p* < 0.05), but no differences were found for vitamin D, CoQ10, and A.

Mineral levels in placenta are shown in Table 4. Mn and Fe were increased in the COVID-19 group (*p* < 0.01), while no differences between groups were found for Se, Ba, Cu, and Zn.

**Figure 2:**
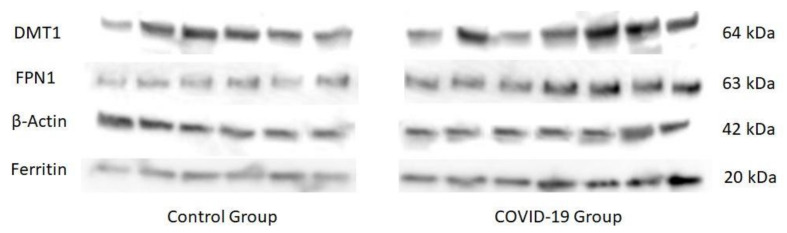
Inmunoblots representing placental protein expression of DMT1, FPN1, β-Actin y Ferritin in healthy and COVID-19 groups of mothers.

**Figure 3:**
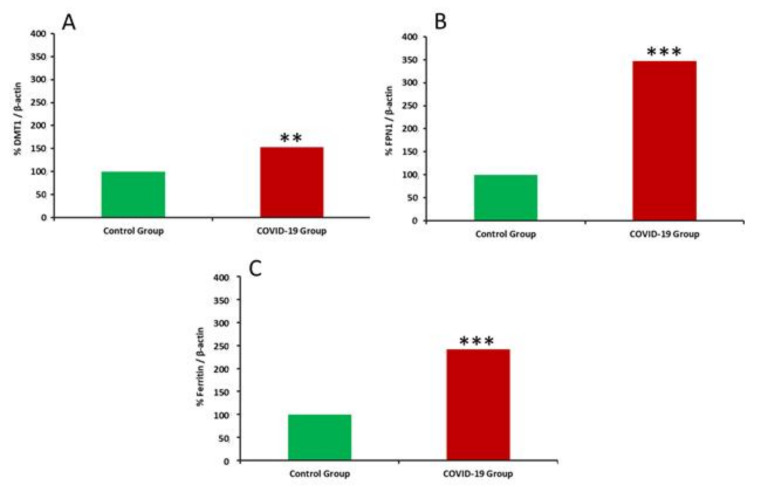
Placental iron metabolism protein expression in healthy and COVID-19 group of mothers. (**A**) Divalent Metal Transporter 1 (DMT1); (**B**): Ferroportin 1; (**C**): Ferritin. Values are expressed as % vs. β-actin. Significantly different from the control group (** *p* < 0.01, *** *p* < 0.001, Student’s *t* test).

Expression of iron-regulatory proteins in placenta is shown in Figure 2 and Figure 3. An up-regulation of placental DMT1, FPN1, and ferritin expression was recorded in placentas of women who suffered the infection compared to healthy women (*p* < 0.01 for DMT1 and *p* < 0.001 for FPN1 and ferritin).

## 4. Discussion

The primary finding of this study was the fact that SARS-CoV-2 infection induces oxidative stress and iron metabolism dysregulation in mother and placenta, highlighting that even in cases of mild infection, COVID-19 is able to induce alterations that can affect the development of the fetus. A major strength of this study was that placental tissue and serum samples were collected from groups with no differences between cesarean and vaginal delivery, which minimized endogenous oxidative stress differences due to labor.

Oxidative stress has a crucial role in COVID-19 [24,25,26]. Infections with RNA viruses, such as respiratory ones, have been linked to an increase in free radical production and antioxidant consumption. SARS-CoV-2 is not an exception, and may cause oxidative stress in the same way that other RNA viruses do [27]. From a physiologic point of view, free radicals provide protection against pathogens [28], but the oxidative stress that accompanies long-lasting viral infections [29] has been associated with impaired immune responses [30,31]. A relation between ROS, endothelial damage, and inflammation has been also reported [32]. This endothelial dysfunction due to ROS production by NADPH-oxidase, lowers the bioavailability of nitric oxide, resulting in vasoconstriction, inflammation and redox imbalance. As a result of viral infection, the renin-angiotensin–aldosterone system becomes dysfunctional and acts as a potent pro-oxidant in blood vessels [33].

This relationship between COVID-19 and oxidative stress is of great importance, especially in situations of high oxidative risk, such as pregnancy or childbirth [10,34], when the production of free radicals is increased due to various reasons and can be the cause of improper embryo implantation, premature birth or malformations, among other aspects [35]. One of the possible free radical sources that has been observed to be altered by COVID-19 is the placenta. The affinity of SARS-CoV-2 for angiotensin-converting enzyme 2 (ACE2) is higher than the SARS-CoV [36] in a pathway that involves the activity of ACE2 cleaving angiotensin II (Ang II), which is previously generated by angiotensin-converting enzyme (ACE). Based on single-cell RNA sequencing analysis, all the trophoblastic cells, which are in direct contact with the maternal blood in the intervillous space, show high expression of ACE2 throughout the whole pregnancy, suggesting that placenta has the potential to be infected by SARS-CoV-2 via a receptor-mediated mechanism, leading to placenta dysfunction and pregnancy complications [37,38].

Placenta plays a crucial role in separating mother and fetus, as well as in regulating the organism’s unique immunological response, allowing a proper embryo implantation and growth, while minimizing the generation of reactive oxygen species (ROS) at this level [35]. However, despite all these effects, the placenta is a great producer of free radicals itself. Organelles abound in trophoblasts’ cytoplasm, which is indicative of the high synthetic and metabolic activity of this unique organ. In addition, placental mass increases throughout healthy pregnancies, which is associated with an augmented mitochondrial activity, one of the main sources of free radicals [35]. As a result, placental tissue is extremely prone to oxidative stress, especially when iron metabolism is impaired. If this situation is not controlled, or normal ROS production gets increased by other factors related to oxidative stress like SARS-CoV-2 infection, damage can have a significant impact on its endocrine and transport activities, as well as cause growth limitation and other development issues [34,35,39]. This could also predispose the offspring to a number of illnesses that may show later in life, with noteworthy consequences for chronic diseases management and public health [40]. This could be the case of our study, which shows how mothers suffering from COVID-19 have a higher oxidative aggression in both placenta and serum obtained during delivery.

Therefore, generated ROS may inactivate biomacromolecules and impair cell metabolism, leading to oxidative stress in the mother, endothelial dysfunction in placenta, and excessive trophoblasts apoptosis. Furthermore, as a consequence of this rise in free radical generation, an increase in anti-angiogenic soluble FMS-like tyrosine kinase-1 (sFlt-1) has been observed, neutralizing circulating endothelial growth factor (VEGF) and impairing the formation of new placental vessels and the placenta function, facts which are associated with preeclampsia and intrauterine growth restriction [41,42,43]. Moreover, oxidative stress combined with insufficient scavenging systems, as measured in the current study, can result in vascular aging [44]. Lipid peroxidation is also linked to atherogenic processes and pathological angiogenesis [45]. In this sense, during intrauterine growth restriction, vascular processes similar to atherosclerosis have been observed in the placenta, which is characterized by arterial vasoconstriction, endothelial dysfunction, and increased oxidative stress [46].

As previously commented, oxidative damage gets aggravated when alterations in iron metabolism are also present [47]. Iron is transported across the membrane of throphoblasts via the divalent metal transporter DMT1 and released from the basolateral membrane to the bloodstream via FPN. Excess of intracellular iron is stored in ferritin. Oxidation of Fe^2+^ to Fe^3+^ is critical for safe iron storage and is mediated by H-ferritin, which possesses ferroxidase activity. The lysosomes recycle iron from mitochondria and cytosolic ferritin. Due to the acidic and reductive environment, lysosomal iron is mostly present in the Fe^2+^ form and is potentially redox-active and the impairment can result in oxidative stress [48]. In our study, mothers who suffered SARS-CoV-2 infection showed a noteworthy increased upregulation of DMT1, FPN1, and ferritin in trophoblasts, which leads to higher iron uptake, storage, and placental sequestration, contributing to the fall in serum iron concentrations [49], a fact that contributes to the evoked oxidative stress in placenta and is consistent with an acute phase response [50,51]. Ferritin overexpression in placenta of mothers who suffered from COVID-19 could be due to the over-activation of T lymphocytes and the over-activity of IFN-γ during the inflammatory process. It has been previously reported that ferritin is related to macrophage activation [52], and COVID-19 patients show high ferritin levels correlated to disease severity [53]. Besides an active secretion during an inflammatory response, ferritin can also induce cell death, and after release, produce extremely high levels of free iron [54], which could explain the evoked oxidative stress in infected mothers. Free iron excess during inflammatory processes enhances the inflammatory reaction, inducing a procoagulant state [54]. Additionally, the release of iron into the bloodstream during COVID-19 infection mediates Fenton and Haber-Weiss reactions, causing oxidative stress due to free Fe (III) ions [55], which also increases ferritin levels [56]. These processes could be explained by the increased FPN1 expression recorded in the study. Moreover, SARS-CoV-2 also features hepcidin-mimic effects, which result in increased ferritin levels [13]. As a consequence, the virus-mediated iron dysregulation contributes to oxidative stress, ferroptosis, lipid peroxidation, and mitochondrial damage, among others [13].

As for antioxidant vitamins, an increase in vitamin D and Coenzyme Q10 (CoQ10) was recorded in the placenta of women suffering from COVID-19, together with an increase in vitamin E in both placenta and plasma of these women. Vitamin D upregulates the expression of some antioxidant genes, such as glutathione reductase, reducing the amount of ROS generated during inflammatory signaling. These free radicals are believed to contribute to the tissue damage related to the development of acute respiratory viral infections, so vitamin D activity would be beneficial in order to avoid this damage [57]. In addition, vitamin D has a key role in the normal development of antigen-presenting cells [58], influencing macrophages and dendritic cell activity. In contrast, vitamin D deficiency impairs innate immune function [59]. This vitamin can also promote antiviral immunity, which is of great importance in SARS-CoV-2 infection. In this sense, it is related to several mechanisms, such as the induction of cathelicidin and defensins, which can block viral entry into cells as well as suppress viral replication [60]. On the other hand, vitamin D promotes autophagy [61], which is a key mechanism to deal with viral infections. Autophagic encapsulation of viruses packages them for lysosomal degradation, antigen-presentation, and subsequent activation of adaptive antiviral immune responses [62]. The specific mechanisms by which vitamin D promotes autophagy involve the downregulation of the mTOR pathway, which inhibits autophagy [63], and the promotion of Beclin 1 and PI3KC3, key enzyme drivers of autophagy [64]. This vitamin is also able to stimulate the formation of autophagosomes through induction of cathelicidin expression, which in turn stimulates key autophagy factors like Beclin 1 [65].

Other important components of the antioxidant defense system that have been studied are selenium, copper, and zinc, as well as vitamin E, which is a major protective lipophilic antioxidant against oxidative stress during pregnancy [66]. Deficits in any of these nutrients influence immunological responses and virus pathogenicity, according to epidemiological research. Even though there were no differences in selenium, copper, and zinc, vitamin E reported to be increased in both placenta and plasma of COVID-19 group. This vitamin works through antioxidant mechanisms that help boost T cell counts, improve mitogenic lymphocyte responses, and raise IL-2 cytokine release and ameliorate NK cell activity, thus reducing the risk of infection. This is the reason why vitamin E supplementation has been found to improve resistance against respiratory infections [67,68,69]. On the other hand, CoQ10 plays a key role in both production of energy and formation of signaling ROS [70], but as soon as ROS levels get excessively increased, it acts as a powerful antioxidant, either directly [71] or via the regeneration of vitamin E [72]. The higher levels of CoQ10 found in the placenta of the mothers who suffered from COVID-19 are correlated with the increased levels of vitamin E also found in this group of women. We hypothesize that the increase in vitamin D, E, and CoQ10 in placenta is a result of a higher uptake of these vitamins in the trophoblast cells so as to cope with the evoked oxidative stress generated in the viral infection.

The imbalance in antioxidant/pro-oxidant status and the iron metabolism impairment recorded in the current study could lead to functional and structural alterations in placental mitochondria [40], together with damage to trophoblast cells. Furthermore, this situation can produce several pregnancy complications related to endothelial cell dysfunction, resulting in systemic endovascular inflammation that would cause symptoms like hypertension and pre-eclampsia [73]. Placental excessive oxidative stress is also associated with the impairment of transport mechanisms in trophoblastic cells [74], decreasing angiogenesis and therefore leading to adverse fetal development [75].

This study presents several strengths and some limitations that should be taken into consideration. With regard to its strengths, according to our knowledge, this is the first study evaluating the influence of COVID-19 on oxidative stress and iron metabolism of pregnant women’s placenta and plasma. Its results will help deepen the knowledge of the effects that SARS-CoV-2 infection can cause in mothers during pregnancy, as well as understand possible subsequent clinical manifestations that could affect the mother and her offspring. Another strength in this study has been the recruitment, sampling, and processing process during the most critical months in the pandemic, dealing with a complex situation in saturated hospitals. The difficulty of getting these risky samples has permitted the gathering of more information on SARS-CoV-2-induced tissue changes and its consequences. Finally, the participation of three different hospitals in two different Spanish regions deserves to be mentioned, having involved more than 20 healthcare professionals, including gynecologists, pediatricians, nurses, and midwives.

Likewise, the study shows some limitations that could be mentioned. A larger sample size would have led to more solid evidence and probably more statistically significant differences in some parameters. The inclusion of an international hospital would have allowed more complete sociodemographic features in the sample of study, covering a more heterogeneous population. It would have been desirable to study iron metabolism in neonates in order to understand the full extent to which the observed maternal and placental outcomes may affect the newborn, because, a possible effect of the alterations observed in placenta and maternal serum iron metabolism could induce in the newborn the development of anaemia, in a more or less severe degree. This should be considered in further studies. Finally, a subsequent monitoring of subjects would have provided more information about the evolution of the disease and possible non-immediate complications derived from it.

## 5. Conclusions

**Figure 4:**
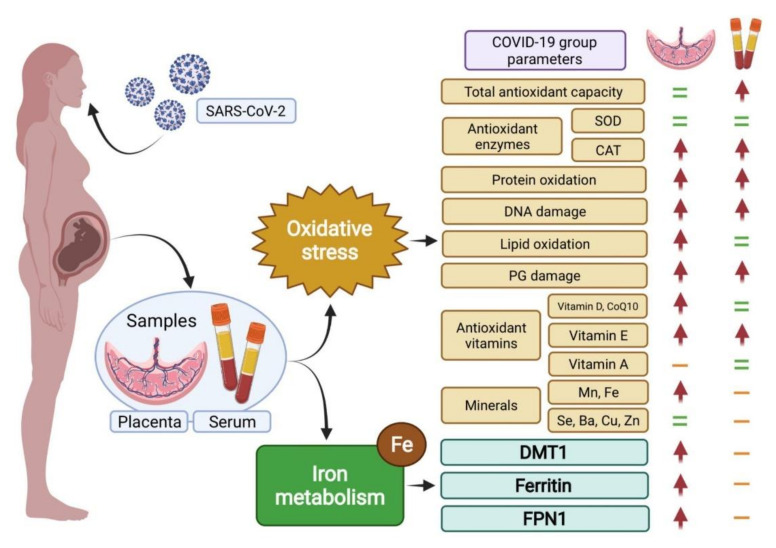
Main attained results on pregnant women related to oxidative stress and iron metabolism after SARS-CoV-2 infection.

In conclusion, our results (Figure 4) suggest, for the first time, an association between COVID-19, placental iron metabolism impairment, and oxidative stress. The overproduction of ROS and impairment in the antioxidant system could accelerate the premature aging of the placenta, also inhibiting cell proliferation in it. COVID-19 would also induce damage to proteins, lipids, and DNA in trophoblasts. An increase in vitamin D, E, and CoQ10 in placenta was observed. This could be a result of a higher uptake in trophoblast cells in order to cope with the evoked oxidative stress related to viral infection. Due to the potential role of oxidative stress for generating abnormalities in placental function and pregnancy complications, further research is needed so as to explain the pathological mechanisms of COVID-19 that affects pregnancy, thus assessing the short-term and long-term outcomes in the mothers’ and infants’ health.

SOD, superoxide dismutase; CAT, catalase; PG, prostaglandins; CoQ10, coenzyme Q10; DMT1, divalent metal transporter 1; FPN1, ferroportin 1. Red arrow: differences were observed between healthy and COVID-19 groups. Green equals sign: no differences were observed. Orange dash: not determined in this sample.

## Figures and Tables

**Table 1:** Clinical characteristics of healthy mothers (*n* = 61) and those who suffered COVID-19 (*n* = 63).

	Control	COVID-19
Age (years)	31.58 ± 1.09	31.96 ± 0.78
Weight (kg)	73.69 ± 2.55	74.12 ± 2.67
Length (cm)	166.83 ± 1.03	163.97 ± 0.68
BMI (kg/m^2^)	26.31 ± 1	27.3 ± 0.8
Parity	Uni (%): 52.22	53.14
	Multi (%): 47.28	46.86
Delivery method	V (%): 56.2	58.7
	A (%): 21.8	19.9
	C (%): 21.8	23.8
Hemoglobin 2nd T (g/L)	11.88 ± 0.23	11.53 ± 0.13
Hemoglobin 3rd T (g/L)	11.96 ± 0.23	11.72 ± 0.17
Hematocrit 2nd T (%)	35.32 ± 0.61	34.11 ± 0.37
Hematocrit 3rd T (%)	35.70 ± 0.63	34.82 ± 0.47
Serum Iron 3rd T (µg/dL)	97.05 ± 14.35	60.14 ± 9.87 **

BMI: Body mass Index; T: Term; V: Vaginal; A: Assisted Vaginal Delivery; C: Cesarean. ** Significantly different from the control group (*p* < 0.01, Student’s *t* test).

**Table 2:** Oxidative/antioxidant parameters in placenta and serum of healthy mothers (*n* = 61) and those who have suffered COVID-19 (*n* = 63).

	Placenta		Serum	
	Control	COVID-19	Control	COVID-19
ABTS (mmol/L Trolox)	3.09 ± 0.15	3.11 ± 0.11	3.34 ± 0.175	2.78 ± 0.161 *
SOD (mU/mg protein)	440.02 ± 40.14	430.25 ± 30.69	4.45 ± 0.43	3.92 ± 0.29
CAT (mU/mg protein)	186.18 × 10^3^ ± 9.28	168.72 × 10^3^ ± 5.10 **	77.01 ± 5.90	62.86 ± 2.86 *
8-OhdG (ng/mL)	283.70 ± 7.98	316.12 ± 6.95 **	75.82 ± 2.85	82.38 ± 1.51 **
Hydroperoxides (µM)	40.73 ± 2.47	48.02 ± 1.90 ***	1.51± 0.27	1.12 ± 0.20
Isoprostanes (ng/mL)	35.30 ± 1.31	39.85 ± 0.57 **	4.35 ± 0.87	8.85 ± 0.22 **
Carbonyl groups (nmol/mg protein)	12.74 ± 0.67	16.37 ± 0.84 ***	1.385 ± 0.026	1.601 ± 0.054 *

Data are shown as the mean values ± SEM. Significantly different from the control group (* *p* < 0.01, ** *p* < 0.01, *** *p* < 0.001, Student’s *t* test).

**Table 3:** Antioxidant vitamins in placenta and serum of healthy mothers (*n* = 61) and those who have suffered COVID-19 (*n* = 63).

		Control	COVID-19
Placenta (µg/mg protein)	Vitamin D	12.45 ± 0.58	14.06 ± 0.51 **
	Vitamin E	64.80 ± 6.87	93.47 ± 7.27 **
	Coenzyme Q10	82.09 ± 4.06	92.13 ± 2.84 *
Serum (µmol/L)	Vitamin D	46.35 ± 2.59	53.22 ± 3.00
	Vitamin E	23.10 ± 2.35	29.47 ± 1.59 *
	Coenzyme Q10	0.44 ± 0.02	0.48 ± 0.03
	Vitamin A	2.29 ± 0.20	2.72 ± 0.28

Data are shown as the mean values ± SEM. Significantly different from the control group (* *p* < 0.05, ** *p* < 0.01, Student’s *t* test).

**Table 4:** Minerals in placenta of healthy mothers (*n* = 61) and those who have suffered COVID-19 (*n* = 63).

	Control	COVID-19
Mn (µg/g DM)	3.93 ± 0.92	7.47 ± 1.07 **
Se (µg/g DM)	7.47 ± 0.63	7.40 ± 0.64
Ba (µg/g DM)	0.47 ± 0.07	0.36 ± 0.03
Cu (µg/g DM)	8.66 ± 0.57	8.72 ± 0.38
Zn (µg/g DM)	49.18 ± 2.18	49.62 ± 1.51
Fe (mg/g DM)	3.54 ± 0.40	5.39 ± 0.45 **

DM: dry matter; Data are shown as the mean values ± SEM. Significantly different from the control group (** *p* < 0.01, Student’s *t* test).

## Data Availability

All of the data is contained within the article.
